# A rare case of splenobronchial fistula formation post-embolisation therapy following acute blunt trauma

**DOI:** 10.1259/bjrcr.20160100

**Published:** 2016-11-07

**Authors:** Saminderjit Kular, Tim Hoare, Colin Nice

**Affiliations:** Newcastle Upon Tyne Hospitals NHS Foundation Trust, Newcastle Upon Tyne, UK

## Abstract

We present the case of a 26-year-old male who was referred to the Emergency department with frank haemoptysis, fever and abdominal pain. He had suffered from an acute splenic rupture secondary to blunt abdominal trauma 3 weeks previously, when he was treated with transfemoral embolisation therapy. On this previous admission his splenic injury was initially not detected owing to CT scanning technique focussed on imaging the thorax rather than the abdomen. On readmission, the initial chest X-ray pointed towards a likely pneumonia or empyema. However, upon CT scanning, the cause was found to be a splenic abscess that had extended through the diaphragm, pleura and entered the bronchial space, forming a ‘splenobronchial’ fistula. This is a rare complication of splenic artery embolisation. The aim of this case report is to raise awareness of the complications that acute trauma and embolisation therapy may cause.

## Clinical case presentation

A 26-year-old male presented to Accident and Emergency following assault with a baseball bat. His past medical history consisted of longstanding Hepatitis C infection only, he was on no regular medications nor had any known allergies.

### Post-injury admission day 1 (AM)

On admission, the patient had complained of bilateral pleuritic pain on inspiration with visible, tender bruising to the posterior thoracic region bilaterally. There were no urinary, bowel or neurological symptoms reported and no open wounds were visible on examination. The anterior abdomen was soft and non-tender to palpation.

**Figure 1.**
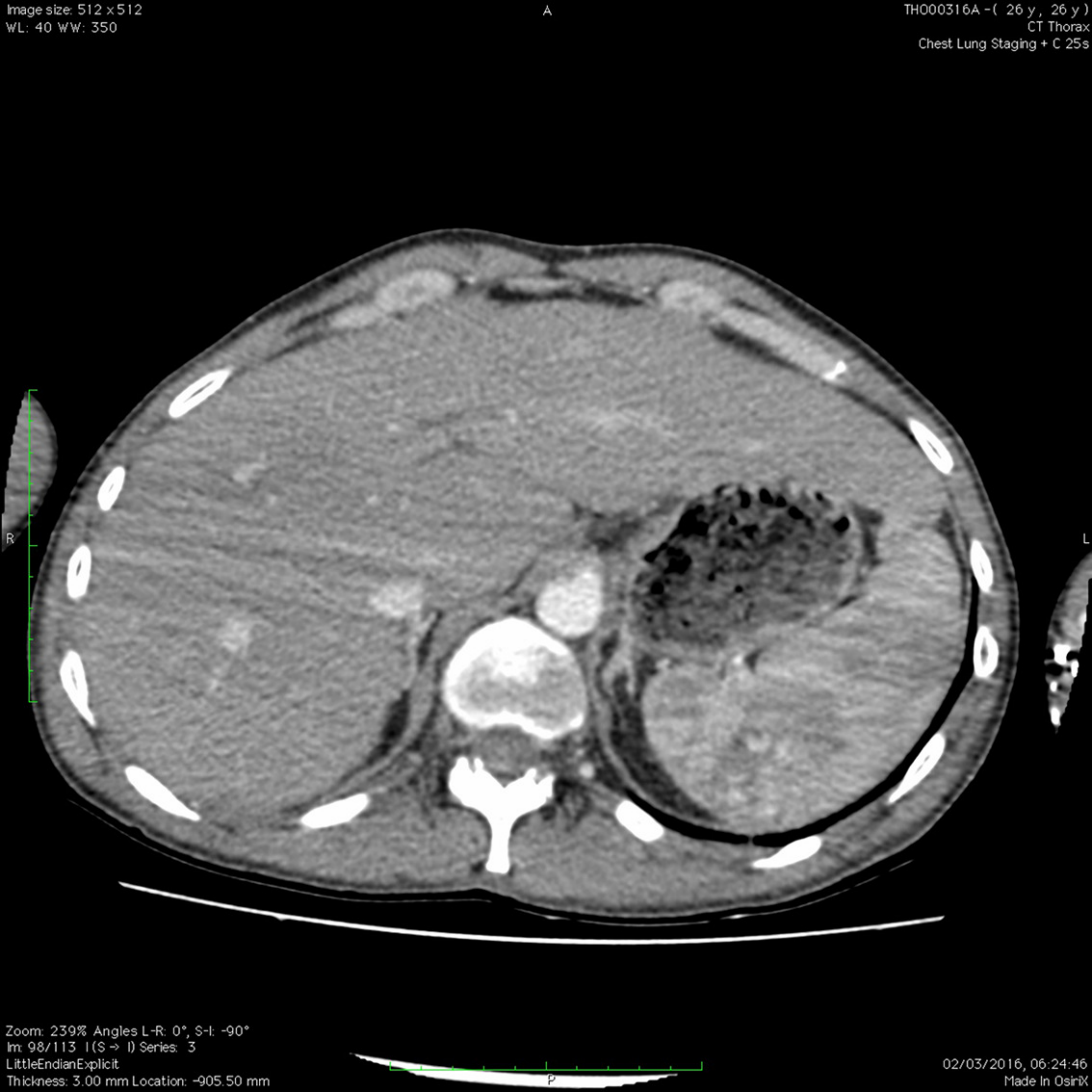
Axial view of the initial CT scan of thorax, acquired at 30s, showing subtle perfusion abnormalities within the spleen.

A CT scan of chest (performed at 30s post i.v. injection) showed acute fractures of left 10th/11thand right 8th/9th ribs, but no evidence of pneumothorax or lung contusion. A splenic laceration was not suspected clinically and the timing of the scan, optimized for the chest, only showed subtle splenic changes which were not identified by the original reporter (Figure 1).

### Post-injury admission day 1 (PM)

**Figure 2.**
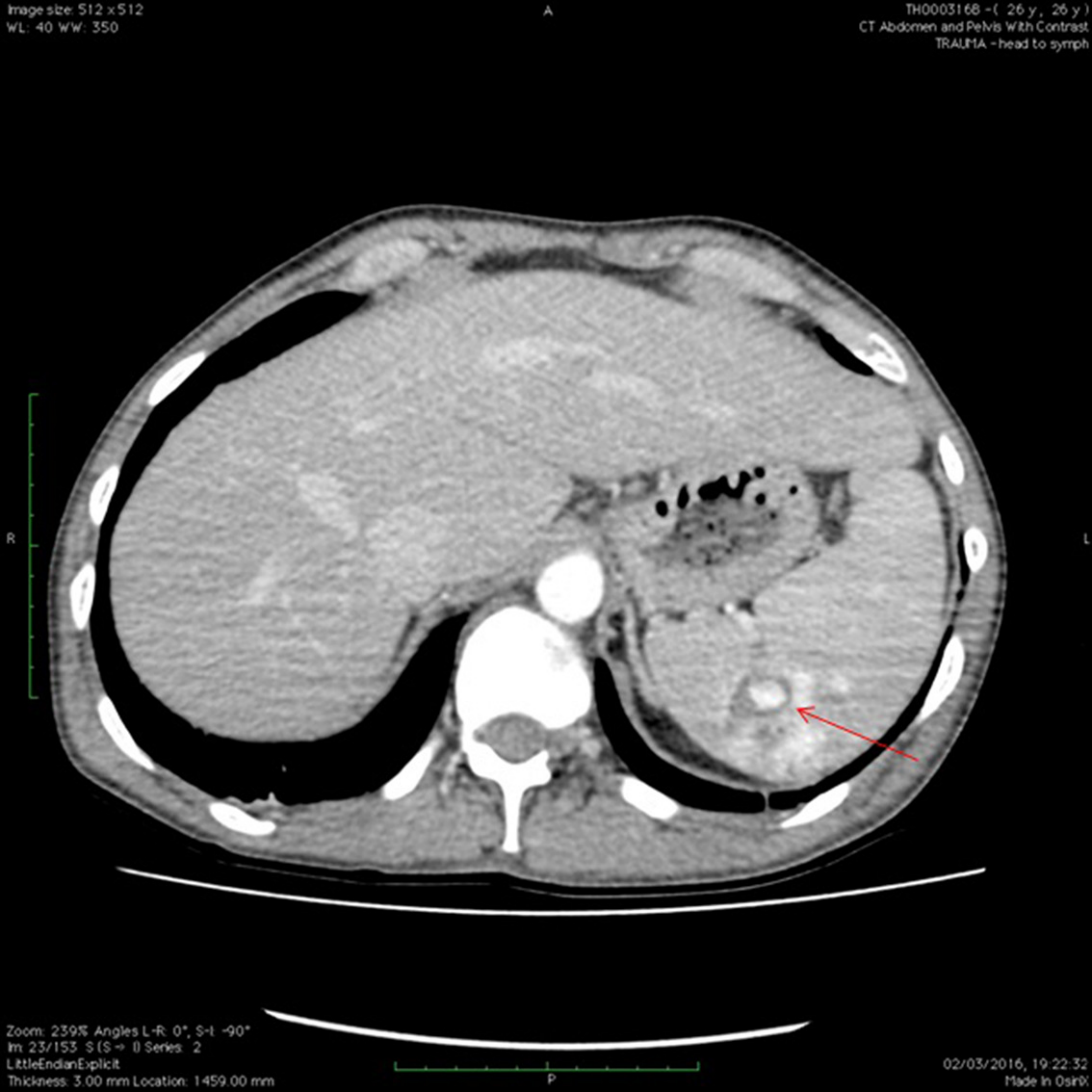
Axial CT scan of abdomen/pelvis with contrast at 70s. Active haemorrhage now visible (red arrow) within the spleen indicating acute injury.

Later that day, the patient complained that their pain had spread from the chest down to the right and left flanks. This prompted a CT scan of abdomen/pelvis (performed at 70 s), which demonstrated a contained splenic laceration with no evidence of capsular breach (Figure 2). Both the rib fractures and splenic injury were treated conservatively with bed rest and analgesia.

### Post-injury day 5

**Figure 3.**
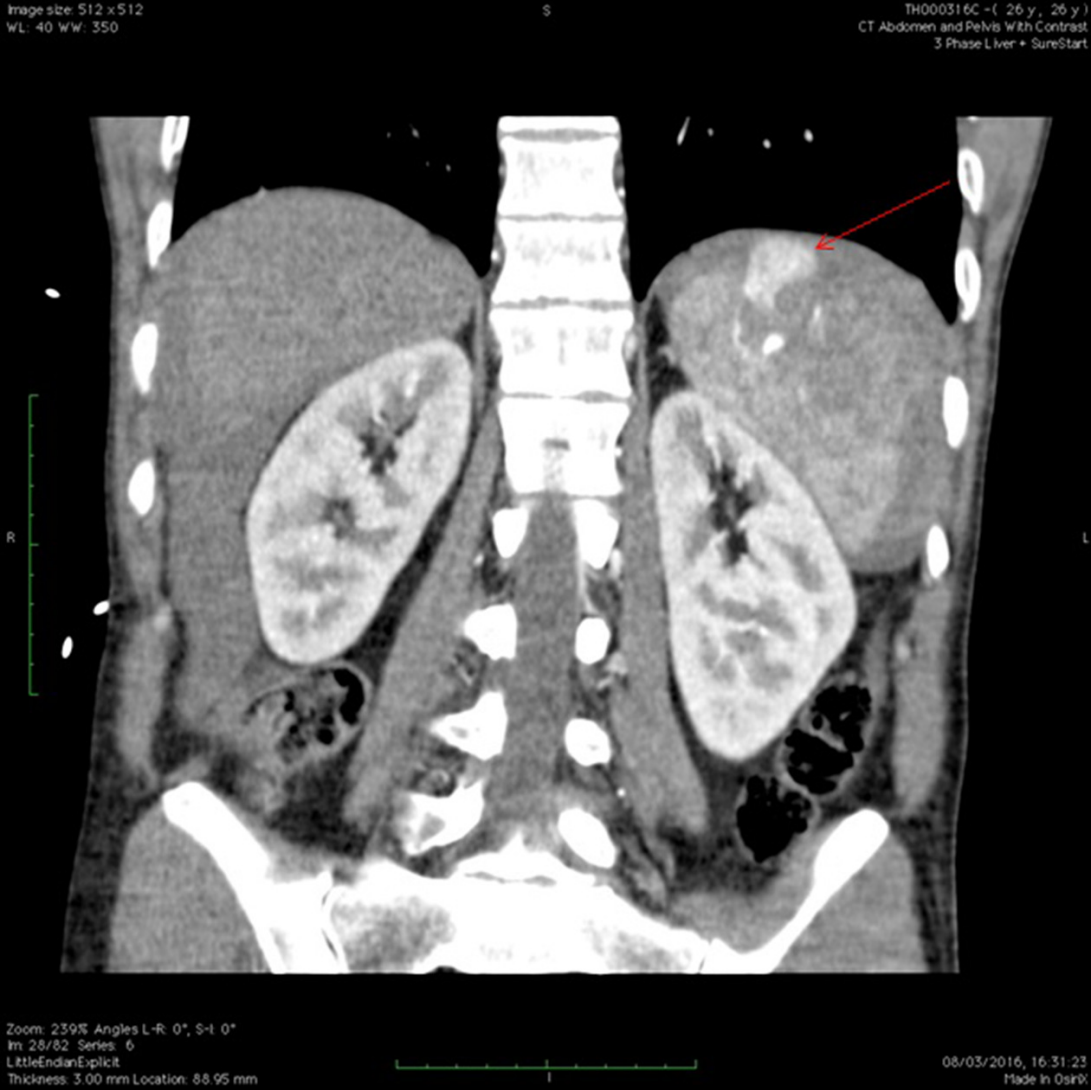
Coronal view of repeat CT scan of abdomen/pelvis with contrast. Active bleeding was still seen at the spleen; however, there was now a new extravasation of contrast (red arrow), indicating splenic capsular rupture.

The patient abruptly deteriorated becoming hypotensive and tachycardic in keeping with hypovolaemic shock. A further CT scan showed splenic capsular rupture, active bleeding and large-volume intraperitoneal haemorrhage (Figure 3).

This was managed urgently with endovascular embolisation. While the patient gave informed consent for the procedure he indicated that he was unlikely to be compliant with some aspects of his aftercare and likely to self-discharge very soon after completion of the procedure.

**Figure 4.**
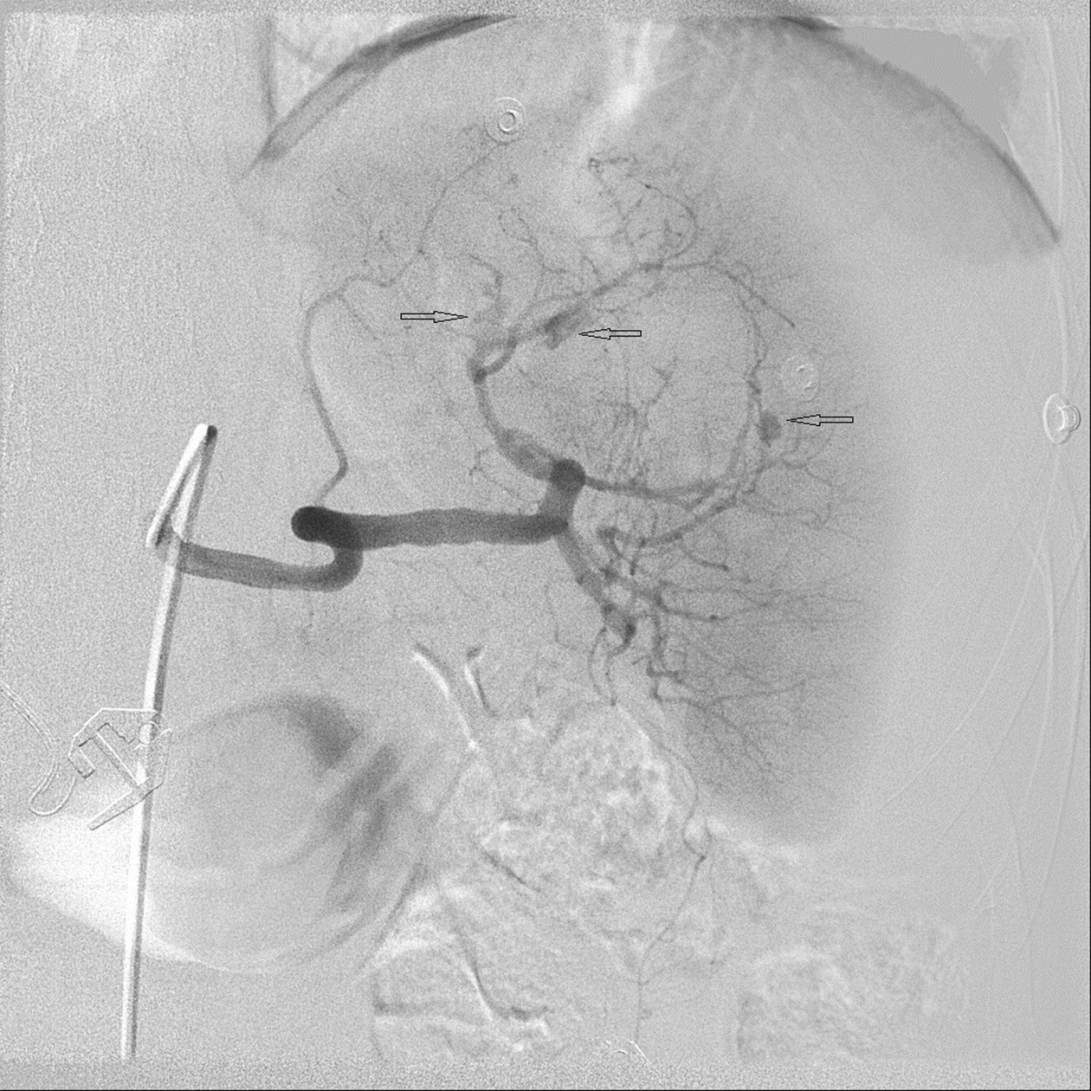
Selective splenic artery angiogram with splaying of the upper pole splenic artery branches owing to haematoma and three pseudoaneurysms (arrows).

Following local anaesthesia an ultrasound guided puncture of the right common femoral artery was performed and the splenic artery selectively catheterized with a Sim 1 catheter (a reverse angled catheter). Angiography revealed three pseudoaneurysms, two arising from a second order upper pole branch and one from a third order equatorial branch (Figure 4). These were then superselectively catheterized and embolized with a series of 3, 4 and 5 mm microcoils (Nester & Vortex, Nester = Cook Medical, Bloomington, IN, USA, Vortex = Boston Scientific, Watertown, MA, USA), delivered through a microcatheter. This abolished filling of the pseudoaneurysms but there was more devascularisation of the upper half of the spleen than envisaged when the decision to perform superselective embolisation was made. The right common femoral artery was then closed with a Starclose vascular closure device (Abbott Medical).

**Figure 5.**
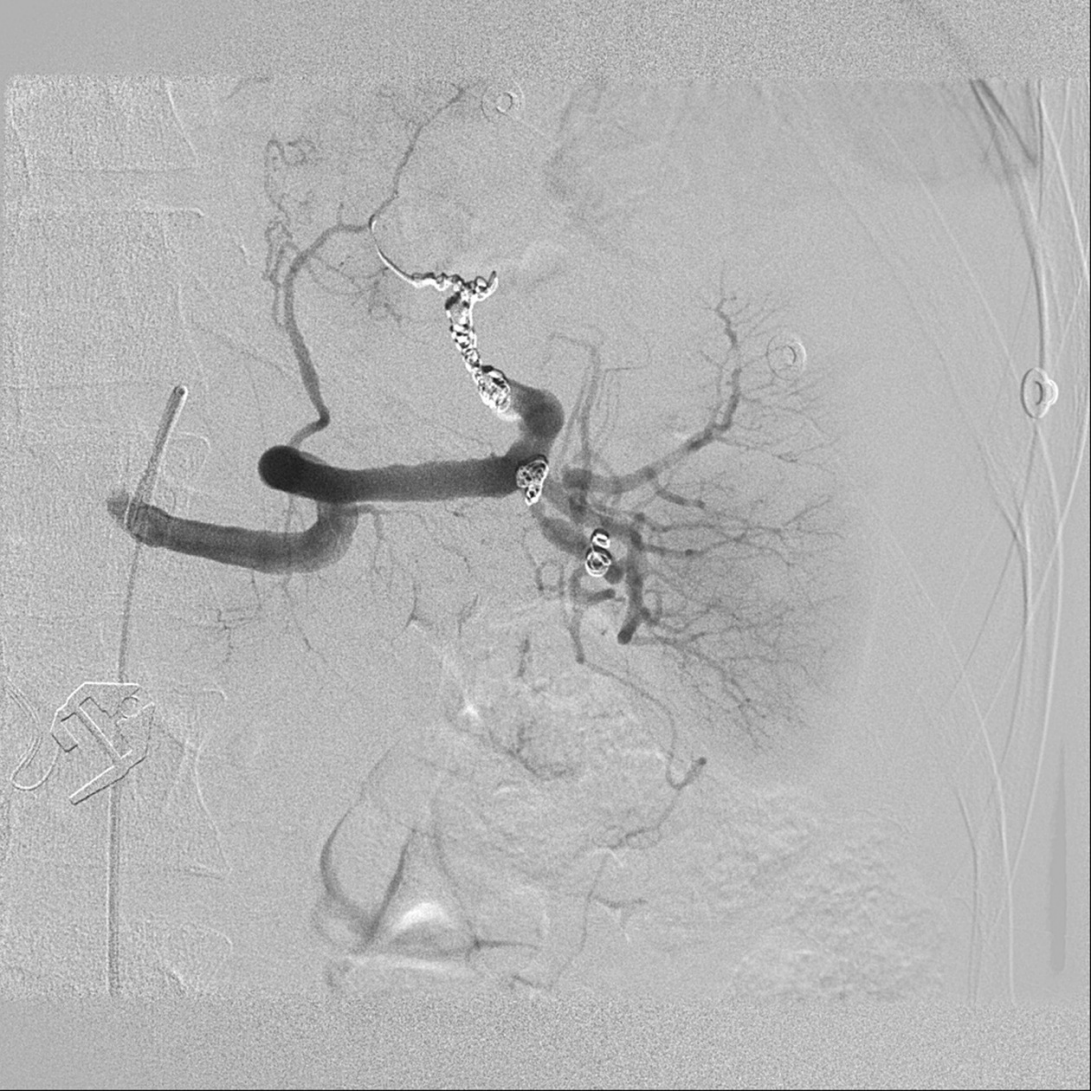
Selective splenic artery angiogram following distal splenic artery embolisation with microcoils (deployed through a microcatheter which was then removed). The upper half of the spleen has little residual arterial filling but the lower half has been preserved.

The decision to perform superselective embolisation was made with the intention of achieving definitive haemostasis in a patient who was likely to leave hospital at the earliest possible opportunity and not comply well with the procedural aftercare (Figure 5). There was no further bleeding.

### Post-injury day 6 (post-embolisation procedure)

Day 1 post-procedure, the patient developed a post-embolisation syndrome (PES) characterized by fever, nausea and left abdominal pain. A fourth CT scan was performed. This showed splenic necrosis, parenchymal and intravascular gas, which is an expected appearance post-embolisation. No fluid collection was demonstrated. Conservative resuscitation was initiated with i.v. fluid and antibiotic administration.

**Figure 6.**
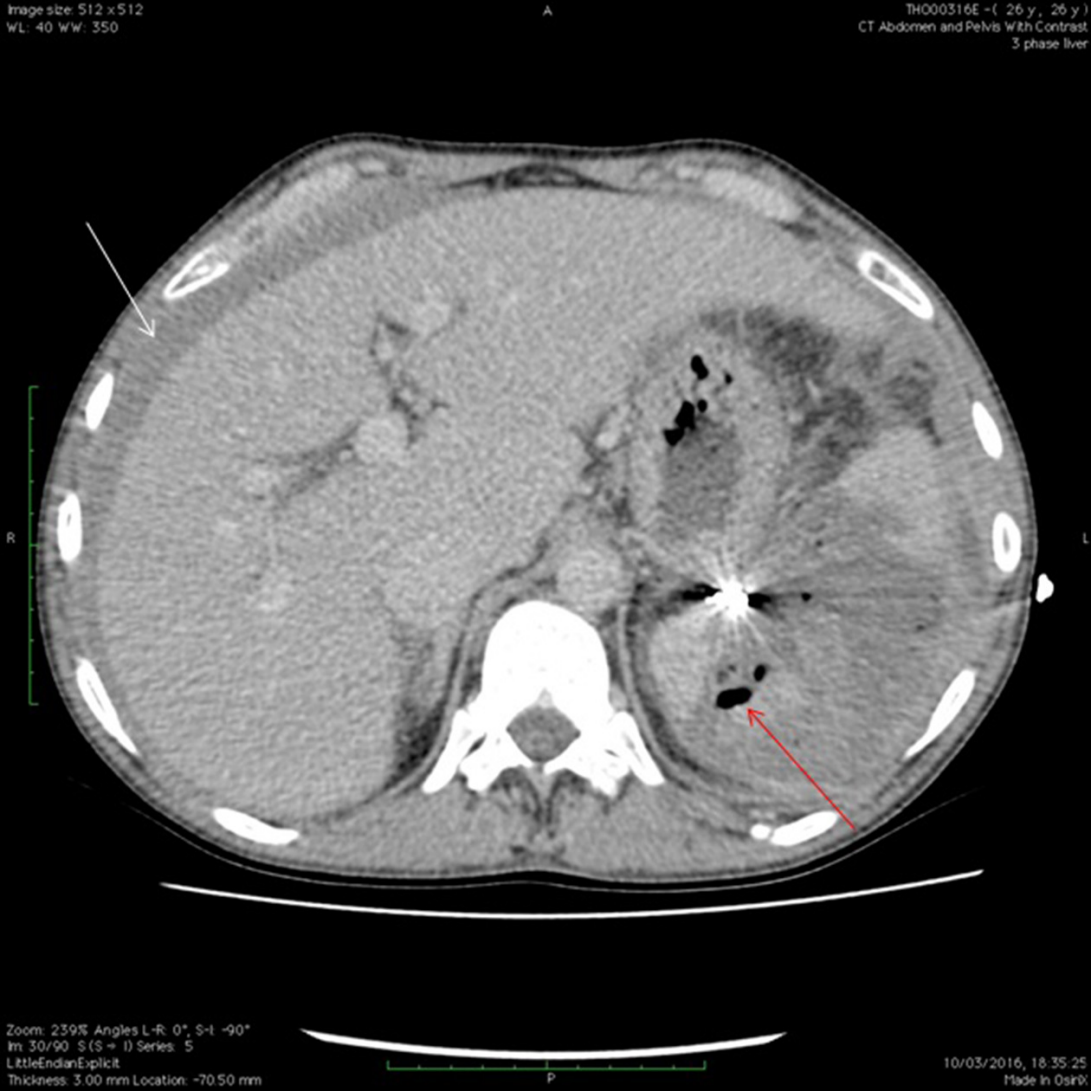
CT scan of abdomen post-embolisation therapy. Embolisation coils noted with a successful cessation of bleeding. Visible gas (red arrow) is seen in the spleen indicating infarction and necrosis. There is also a notable increase in extra-hepatic fluid (white arrow), that likely resulted from delayed presentation following the initial trauma.

There was no indication for surgical drainage at this point as the CT scan showed only splenic necrosis, with no evidence of abscess formation. The patient’s temperature and clinical features were also settling, further reassuring stabilisation of his condition (Figure 6).

The patient recovered from this episode a few days later, and deemed medically fit, was subsequently discharged home.

### Post-injury day 21

3 weeks later, the patient presented to Accident & Emergency once again; this time with episodes of frank haemoptysis. He had an associated fever, and left upper quadrant (LUQ) pain.

Blood tests showed elevated inflammatory markers, suggesting an infection, thought to be of likely respiratory origin at this time.

## Differential diagnosis

The primary clinical diagnosis on readmission was of suspected pneumonia. However, the addition of haemoptysis brought about the suspicion that this could have been possible pulmonary embolism or traumatic arteriovenous fistula formation.

**Figure 7.Chest X-ray:**
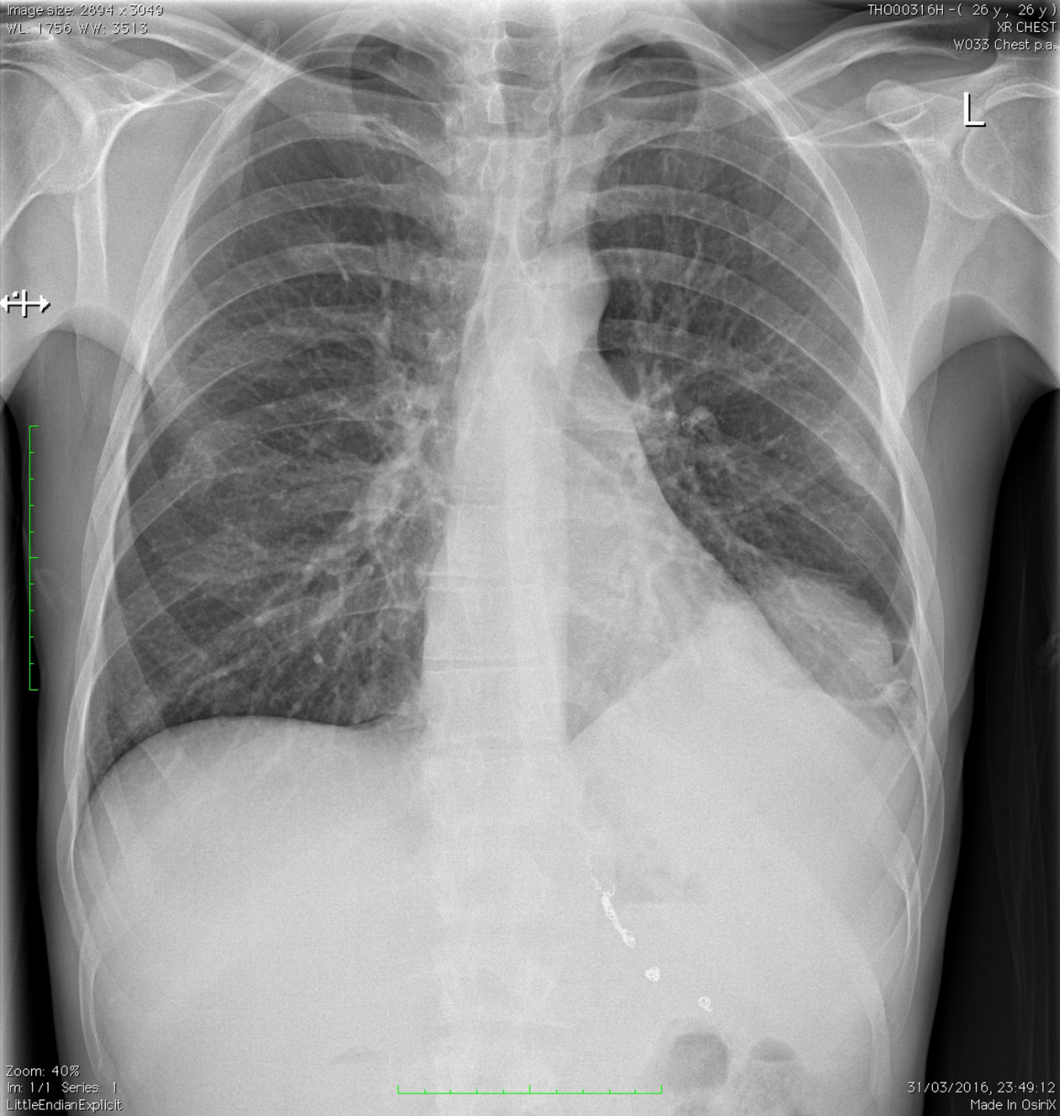
on redmission. Abnormality was noted in the contour of the left diaphragm along with increased opacification of the left lower zone.

Following an initial abnormal chest X-ray (CXR), the suspected diagnosis was revised to possible empyema (Figure 7).

As the patient had associated LUQ pain; it was acknowledged that the spleen could be a potential source of infection, particularly as it is established that splenic abscesses are a known complication of embolisation therapy.

## Investigations and imaging findings

Blood results showed an elevated C-reactive protein and white cell count, consistent with infection. The patient was becoming progressively more breathless and his haemoptysis was at a large volume, approximating 500 ml.

**Figure 8.**
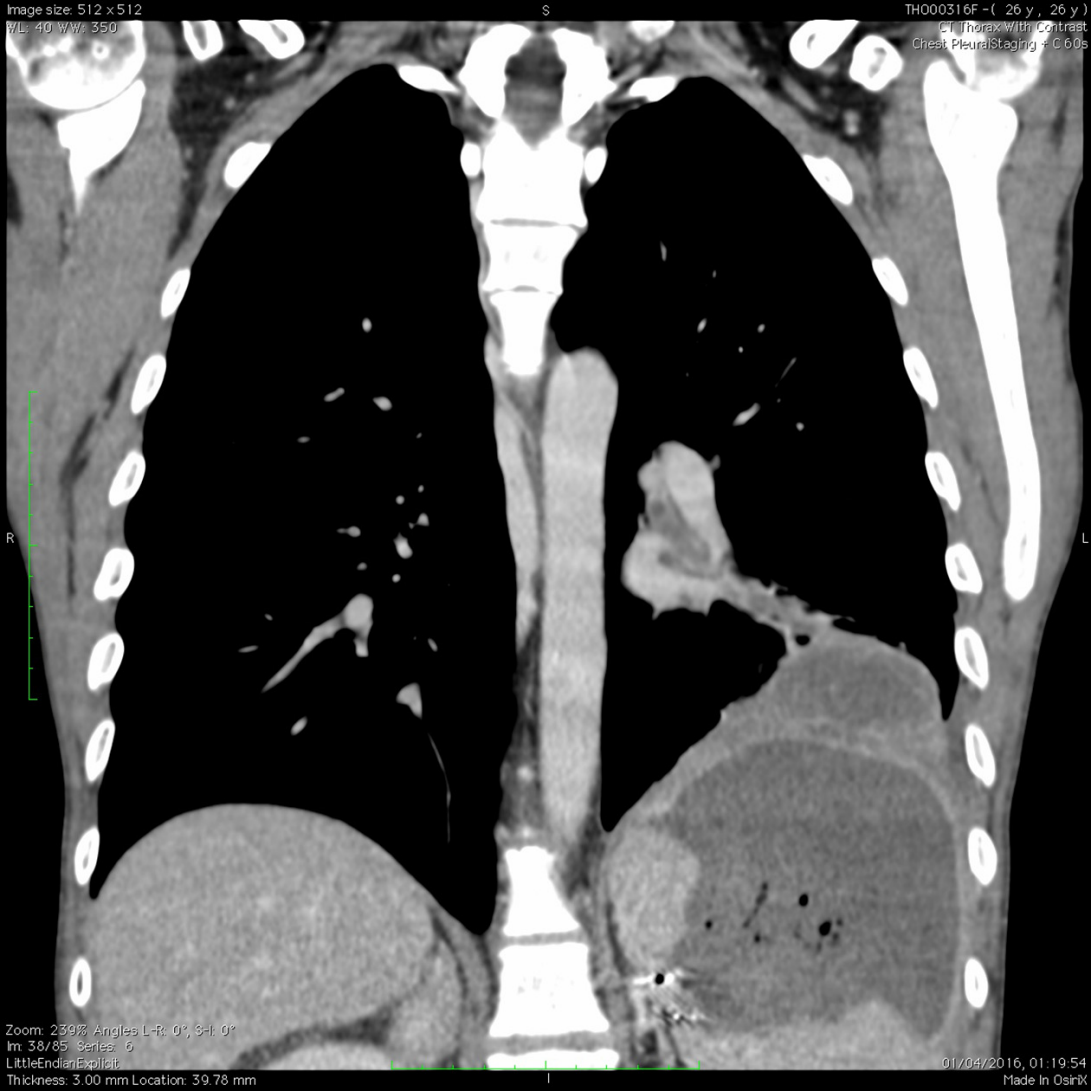
Coronal view of CT scan of abdomen/pelvis with contrast, taken at 60s with Omnipaque 350 agent delivered at 3 ml/s. Demonstration of the splenobronchial fistula; coursing from the spleen via the abscess, into the medial left bronchial region.

A CT scan of thorax/abdomen was requested to clarify this CXR abnormality with a suspicion of underlying empyema. A diagnosis was made of a large splenic abscess, with fistulation through diaphragm, pleura, lung and into bronchi (Figure 8).

A subsequent ultrasound was performed in the context of placing a percutaneous drain. Dynamic images from this showed the fistula across the diaphragm demonstrating a ball-valve effect with infected fluid being drawn into the chest with each respiratory cycle (Supplementary video 1).

## Treatment

In view of the presence of a fistula to bronchus and a diaphragmatic defect, his case was initially discussed with cardiothoracic surgery. However, it was felt that because the source of the problem was intra-abdominal, then it would be most appropriate to address this first.

As a result, the patient was treated via an ultrasound-guided drain, inserted into the splenic cavity. Upon satisfactory positioning of the drain, there was an immediate cessation of haemoptysis and significant improvement of the dyspnoea. 500 ml of thick, infected, necrotic spleen and haemorrhage was aspirated.

## Outcome and follow-up

The drain was left in situ for over 1 month. The patient had most recently undergone a surveillance CT scan 1 month post drain removal, which showed that the splenic collection had resolved and the fistula had healed through scarring.

## Discussion

The overall incidence of splenic abscesses is rare, with a reported frequency of around 0.05–0.7%^1^ Diagnosing a splenic abscess remains a significant challenge; as the typical triad of fever, LUQ pain and splenomegaly is only seen in approximately one-third of patients.

The most common complications following splenic artery embolisation are splenic infarction (3%) and abscess formation (2%).^2,3^ Splenic infarction may give rise to abscess formation.

Minor complications can include atelectasis of the lungs and left-sided pleural effusions; however, these are relatively rare compared to the aforementioned necrotic/infective processes.^4,5^

Splenic artery embolisation may be performed proximally or distally. Proximal embolisation involves occlusion of the main splenic artery with either coils or a vascular occlusion plug to decrease the splenic perfusion pressure but preserve splenic perfusion via the collateral pathways (short gastric arteries, pancreatic arteries, gastroepiploic and splenic capsular arteries). In this technique, the coils or plug are placed in the proximal third of the splenic artery, just distal to the dorsal pancreatic artery.

In contrast distal splenic artery embolisation involves placement of embolic material as close to the vascular abnormality as possible and usually requires a microcatheter system to achieve this.

In several studies comparing proximal to distal embolisation techniques the success rates in stopping bleeding are similar.^6^

There have been inconclusive differences in complication rates of infection and infarction that go on to require radical splenectomy.^7^

Minor complications may be more frequent in distal embolisation procedures, owing to the higher rate of segmental infarctions.^8^ However, as these infarctions tend to be limited to the segment just distal to the site of embolisation, their clinical relevance is questionable.

Proximal splenic artery embolisation is faster, an important consideration in bleeding patients, but may make the distal splenic artery inaccessible to embolisation if rebleeding occurs.

A notable side effect to be aware of in any transarterial embolisation is one of PES. This comprises of symptoms including; fever, nausea/vomiting and pain at the site of injury.^9^ It usually lasts around 72 hours post-procedure and its presentation as a ‘flu-like’ illness can mimic a systemic inflammatory response syndrome, therefore it is important to differentiate PES from true infection so as to guide most appropriate management.^2^ As PES is normally self-limiting, management tends to be conservative with i.v. therapy and analgesia only.

Although many fistulas are theoretically possible through anatomical principle, there have been few recorded events of splenic fistulation in the literature. There has been one previously documented case of similar splenobronchial fistulation, with the remainder comprising largely of the gastrosplenic variety.^10–12^ Thus this case demonstrates a rare phenomenon to have presented in such an atypical way.

The progressive deterioration, along with the corresponding respiratory symptoms, made this an initial diagnostic dilemma.

It was only when imaging was used that the true nature of the pathology was uncovered, highlighting the importance radiological modalities play in modern day diagnosis and management.

## Learning points

1. Keep an open mind in assessing trauma and be aware of the risk of upper abdominal organ injury in the context of chest trauma – performing a trauma protocol of CT scan in such a case would have picked up the initial splenic injury sooner
2. Delayed complications, such as visceral haemorrhage or bowel infarction secondary to mesenteric injury, are recognized in trauma
3. Embolisation can cause organ parenchyma necrosis which causes a systemic response
4. Uncontrolled infection can fistulate into distant anatomical compartments.

## Consent

Fully informed patient consent obtained for publication of all images and clinical information.
